# Prevalence and diversity of gastrointestinal parasites on pig farms in Kalasin Province, Thailand

**DOI:** 10.14202/vetworld.2024.273-281

**Published:** 2024-02-01

**Authors:** Sirikanda Thanasuwan, Supawadee Piratae, Keattipong Thaowandee, Chalothon Amporn

**Affiliations:** 1 Department of Veterinary Technology, Faculty of Agriculture Technology, Kalasin University, Kalasin 46000, Thailand; 2 One Health Research Unit, Mahasarakham University, Maha Sarakham 44000, Thailand; 3 Veterinary Infectious Disease Research Unit, Mahasarakham University, Maha Sarakham, 44000, Thailand

**Keywords:** gastrointestinal parasites, Kalasin, pig, prevalence

## Abstract

**Background and Aim:**

Gastrointestinal (GI) parasite infections are the foremost and prevalent diseases that affect pigs in Thailand. This study aimed to determine the prevalence of GI parasites among pigs in Kalasin Province. This study is the first attempt to understand the occurrence of GI parasites in pigs within Kalasin province using fecal samples as the methodology.

**Materials and Methods:**

We collected 324 fecal samples directly from the rectums of pigs from May to July 2023. The formalin-ethyl acetate concentration and floatation methods were used to examine these samples. The eggs or oocysts were identified by observing their morphology and size under a light microscope.

**Results:**

Of the 324 fecal samples examined, 276 tested positive for gastrointestinal parasitic infections, resulting in a prevalence rate of 85.19%. Nematodes were the most prevalent, with Strongyle-type infections being the highest in 267 cases (82.41%), followed by *Ascaris suum* in 222 cases (68.52%), and *Trichuris* spp. in 152 cases (46.91%). *Strongyloides* spp. infections were also observed in 92 cases (28.40%). However, trematode infection was relatively rare, with only *Fasciola* spp. found in 15 cases (4.63%). We identified *Eimeria* spp. in 87 cases (26.85%), *Iodamoeba* spp. in 70 cases (21.60%), *Balantidium coli* in 67 cases (20.68%), and *Isospora* spp. in 52 cases (16.05%). Notably, most of the positive fecal samples showed double infections with a prevalence rate of approximately 38.27%. Single infections were the next most common, accounting for 25.31% of the cases, followed by 3 parasites (14.81%) and 4–5 parasites (6.79%).

**Conclusion:**

This study underscores the high endemicity of GI parasites among pigs in Kalasin province. To improve prevention and control measures, it is recommended to establish a health monitoring program that includes deworming and emphasizes good hygiene practices. The insights gained from this study will contribute to the enhancement of pig farming practices in Kalasin province, ultimately leading to improved production and profitability. In addition, future research should focus on detecting these parasites in Kalasin and exploring their relationship with human transmission cycle.

## Introduction

Swine production plays a crucial role in the local economies of various regions around the world [1–3]. Pig farming has a dual purpose: increasing the availability of animal protein for human consumption and contributing to poverty reduction. Pig production offers several advantages over other livestock, such as faster and higher returns on investment, early maturity, shorter generation intervals, rapid growth rates, and relatively smaller space requirements [1, 4]. In the last three decades, pig production has undergone significant changes. While there has been a decrease in the number of farms in rural areas, the swine industry has grown and moved toward more intensive production systems. These developments have led to improved hygiene practices and biosecurity measures in the swine industry [5]. Thailand is one of the largest producers of pigs in Asia, and the primary population of native pigs (*Sus scrofa indicus*) is concentrated in remote regions of the north-west highlands [6, 7]. Pork plays a key role as a primary meat source in these areas. In 2017, pork production amounted to approximately 19.5 million pigs, an increase from 180,000 pig farms. Consumption of pork by the Thai population totaled 1.15 million tons [7].

The prevalence and intensity of parasites can vary depending on the type of swine production system [8]. Good hygiene has been shown to reduce the risk of pathogen transmission and larger swine herds have a lower incidence of gastrointestinal (GI) parasites than smaller farms. However, several factors influence the prevalence of parasites in swine farms, such as the type of flooring, bedding materials, and housing facility design [5]. Protozoa, such as *Coccidia* (*Cystoisospora* spp. and *Eimeria* spp.), *Entamoeba* spp., and helminths, are commonly observed in the gastrointestinal tract. The helminths in this category include Strongyle-type species (*Oesophagotomum* spp., *Hyostrongylus rubidus*), *Strongyloides* spp., *Ascaris* spp., *Trichuris* spp., and *Fasciolopsis* spp. [9]. GI parasistes are a significant contributor to economic losses in pig farming. However, farmers may not always recognize their presence because the symptoms often remain subclinical and the swine may appear healthy outwardly [10]. These parasites can lead to clinical gastrointestinal diseases, but more commonly result in subclinical infections that negatively impact weight gain, feed conversion efficiency, growth rates, and overall welfare of the animals [5, 11]. In addition, the condemnation of internal organs, particularly livers with milk spots, caused by the migration of *Ascaris suum* larvae can increase economic losses [5]. *A. suum* can hinder growth and affect cognitive function in children and young adults [12] and is a concern for public health. Infection with *A. suum* can occur by ingesting contaminated food or water containing viable parasite eggs [13]. This parasite has significant health implications and can result in financial losses due to medical treatment [14]. In the context of protozoa, *Coccidia* are important for swine because they are associated with stunted growth and may require the removal of affected pigs from their enclosures for treatment with anti-parasitic drugs. Typically, suckling piglets are administered oral drugs, such as toltrazuril-based coccidiostats, as the primary method for controlling coccidiosis [3].

In rural areas, pigsties are strategically located within residential regions to prevent theft; however, the risk of contamination between pigs and humans, particularly children, is increased, and the potential transmission of virulent zoonotic parasites is also increased [10]. In Thailand, poor pig husbandry can contribute to malnutrition, weaken the immune system, and increase susceptibility to parasites and microbial infections. Consequently, individuals are at risk of parasite contamination when sharing environments such as pastures, food, or water, and some pig parasites may have zoonotic potential [4]. Small-scale farm pig production is usually associated with poor hygiene and low biosecurity between pigs, humans, and wildlife [8]. Small-scale pig farming in Kalasin is often plagued by poor hygiene practices, inadequate deworming, non-standard pigsty conditions, limited access to veterinary and agricultural support services, and insufficient knowledge about parasites. These factors contribute to poor environmental hygiene and poor management of pig farms, which are known risk factors for gastrointestinal parasitic infection. Diagnosis of GI parasites relies on clinical symptoms, fecal analysis, and larval culture to confirm helminth species [15].

This study aimed to provide the first comprehensive report on the prevalence of GI parasites in pigs in Kalasin province through the examination of fecal samples, given the limited reports on GI parasites in pigs in Thailand and the absence of data for Kalasin province.

## Materials and Methods

### Ethical approval

The Institutional Ethical Committee of Kalasin University (KSU-AE-002/2566) supervised and approved this research protocol. All samples were collected without harm to the pigs and the procedures were strictly in accordance with established animal welfare standards and guidelines.

### Study period and location

**Figure-1:**
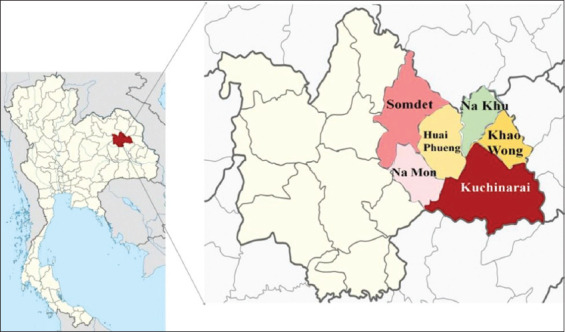
The map of Kalasin province, Thailand (left), and six districts of sampling sites (right) [Source: https://en.wikipedia.org/wiki/Kalasin_Province].

The study was conducted from May to July 2023 on pig farms in the districts of Somdet, Huai Phueng, Na Khu, Kuchinarai, Khao Wong, and Na Mon in Kalasin province, Thailand (Figure-1). These pig farms are located in rural areas because there is animal husbandry. The geographical location of the studied areas is Kalasin Province in North-east Thailand. Kalasin province spans an area of 16.96 km^2^ and is positioned at coordinates of approximately 16° 26’ 3” latitude and 103° 30’ 33” east longitude. The study area was located at an elevation of approximately 147 m above the mean sea level, with an average temperature of 26.8°C and annual rainfall of 1407 mm.

### Sample size

Pig fecal samples were collected randomly from different locations in Kalasin. The sample size was determined using the following equation:


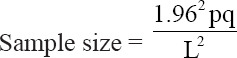


Here, n = sample size, p = expected prevalence, q = 1 − p, and L = limits of error on the prevalence in the local pig population. In view of the fact that the actual prevalence in the local pig population is not known, the calculation was carried out with an assumed prevalence of 75% and a 5% margin of error. The calculated sample size required was 288 pigs [16]. The characteristics of the animals, such as age, gender, and pregnancy status, were not mentioned due to the lack of recording data from farm owners.

### Collection of fecal samples

This study analyzed 324 fecal samples from pigs which were randomly collected from five districts of Kalasin Province. Fresh fecal specimens were directly obtained from the rectum using plastic gloves and carefully placed into appropriately labeled ziplock bags. These samples were immediately stored in an icebox at 4°C for preservation during transportation to the laboratory. Individual fecal samples were processed at the Technology Veterinary Laboratory of Kalasin University upon arrival at the laboratory. Before fecal analysis, samples were refrigerated at a constant temperature of 4°C. The experiment was performed on the same day of stool collection using fecal flotation and the formalin-ethyl acetate centrifugation technique. The fecal examination was conducted using a 10% formalin-ethyl acetate centrifugation technique [17]. Prevalence was calculated using the following equation:


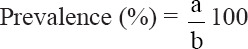


Where, “a” = Number of individuals having a disease at a particular time; “b” = Number of individuals in the population currently at risk [18].

### Parasitological analysis

#### Formaline-ethyl acetate concentration technique

Two grams of feces were combined with approximately 15–20 mL of normal saline, and the mixture was thoroughly vortexed to dissolve the feces. Each dilution was filtered through a wet gauze placed in a funnel into a 15 mL plastic conical centrifuge tube. The tube was then centrifuged at 400× *g* for 3 min. After centrifugation, the supernatant was carefully discarded, 7 mL of 10% formalin solution was added, and the mixture was thoroughly mixed. 3 mL of ethyl acetate was added to this mixture, and the tube was shaken vigorously for 1 min. The tube was then centrifuged again at approximately 400× *g* for 3 min. On completion of centrifugation, three distinct layers could be observed at the top of the tube: An upper layer consisting of ether, a plug containing fecal debris and formalin, and a concentrated sediment containing egg parasites at the bottom. A stick was used to dislodge the layer of fecal debris adhering to the side of the tube, and the ether layer was discarded. The remaining sediments were mixed using an autopipette, and one drop of this mixture was added to a drop of 0.85% Sodium chloride (NaCl) on a glass slide. The slide was covered with a coverslip and subjected to microscopic examination to detect the presence of heavy egg parasites [18].

### Fecal flotation method

Approximately 1–2 g of feces was combined with 3 mL of floatation fluid in a 12 mL test tube and mixed thoroughly to process the fecal sample. A 33% NaCl solution was used as the floatation fluid in this case. By gently stirring, sufficient floatation fluid was added to create a visible meniscus at the top edge of the test tube. It allowed small egg parasites or oocysts to float to the surface. A cover slide was carefully placed on top of the floated material for microscopic examination after approximately 15 min.

### Statistical analysis

Prevalence was determined by dividing the number of positive samples by the total number of samples tested.

## Results

**Figure-2:**
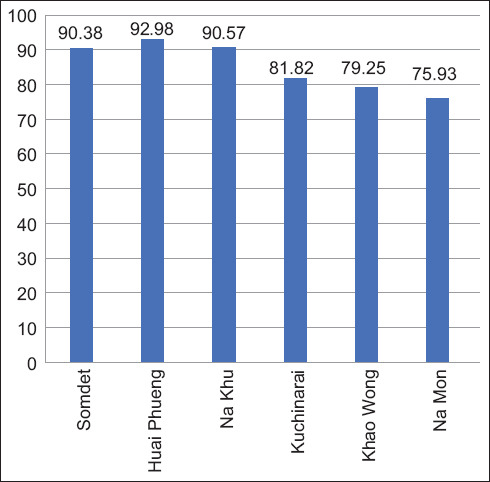
Prevalence of gastrointestinal infection by genera in pigs in Kalasin province (6 districts), Thailand.

**Figure-3:**
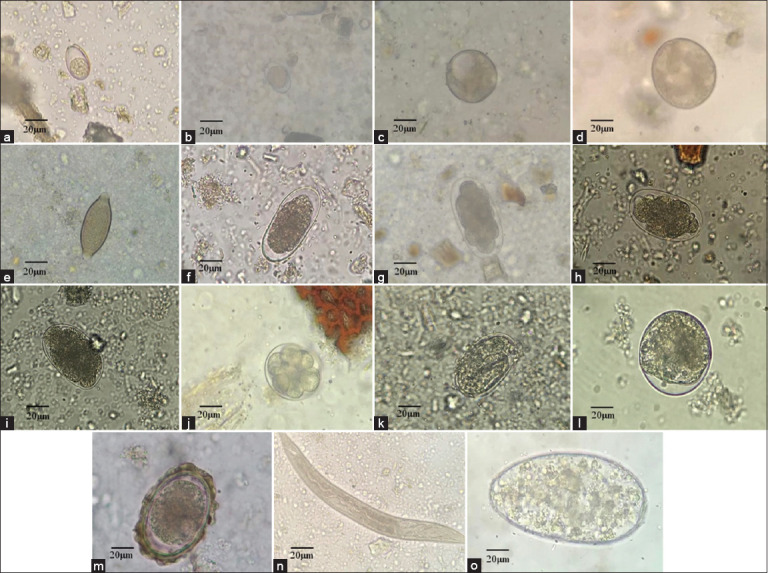
Gastrointestinal parasite present in pigs samples. (a) *Eimeria* spp.; (b) *Isospora* spp., (c) *Iodamoeba* spp., (d) *Balantidium coli*, (e) *Trichuris* spp.; (f-j) Strongyle-type; (k) *Strongyloides* spp.; (l-m) *Ascaris suum*; (n) *Strongyloides* spp. (worm); and (o) *Fasciola* spp.

Of the 324 fecal samples collected from pigs in Kalasin Province, Thailand, from April to July 2023, 276 samples tested positive for at least one GI parasite, resulting in an overall prevalence rate of 85.19%. Samples were collected from six districts (Somdet, Huai Phueng, Na Khu, Kuchinarai, Khao Wong, and Na Mon), and GI parasites were observed across all these regions. The infection rate was highest in Huai Phueng district, reaching 92.98% (Table-1 and Figure-2). The analysis identified nine distinct parasite species, including four nematodes, one trematode, and four protozoans. Among the nematodes, Strongyle-type parasites were the most prevalent, accounting for 82.41% of the infections, followed by *A. suum* (68.52%), *Trichuris* spp. (46.91%), and *Strongyloides* spp. (28.40%). Trematode infections, specifically *Fasciola* spp., are comparatively rare, with a prevalence of 4.63%. Protozoan oocysts (*Eimeria* spp., 26.85%, *Iodamoeba* spp., 21.60%, *Balantidium coli*, 20.68%, and *Isospora* spp., 16.05%) were also detected (Table-2 and Figure-3). The prevalence of Strongyle-type parasites was consistently high in all the districts. *A. suum* had the highest prevalence in both Huai Phueng and Kuchinarai (approximately 48%–49%). *Trichuris* spp. had the highest prevalence in Huai Phueng (87.72%), while *Fasciola* spp. had the highest prevalence in Huai Phueng (14.04%). *Strongyloides* spp. and *Eimeria* spp. had the highest prevalence at 58.49% and 47.17% in Na Khu and Khao Wong, respectively. *Isospora* spp., *Iodamoeba* spp., and *B. coli* were most prevalent in Na Mon (27.78%), Huai Phueng and Kuchinarai (32.73%–33.33%), and Huai Phung and Khao Wong (26.32%–26.42%), respectively. Furthermore, most positive fecal samples exhibited double infections, representing the highest prevalence rate at approximately 38.27%, followed by single infections (25.31%, 14.81%, and 6.79%, respectively, Table-3).

**Table-1:** Prevalence of gastrointestinal infection by genera in pigs in Kalasin province, Thailand (six districts).

District	Number of animals	Number of positive	Prevalence (%)
Somdet	52	47	90.38
Huai Phueng	57	53	92.98
Na Khu	53	48	90.57
Kuchinarai	55	45	81.82
Khao Wong	53	42	79.25
Na Mon	54	41	75.93
Total	324	276	85.19

**Table-2:** Prevalence and distribution of gastrointestinal parasites in farm pigs in Kalasin, Thailand.

Parasites		Prevalence (%) (n = 324)	District					
	
Phylum	Name of parasite		Somdet (n = 52)	Huai Phueng (n = 57)	Na Khu (n = 53)	Kuchinarai (n = 55)	Khao Wong (n = 53)	Na Mon (n = 54)
Nematodes								
	Stronglyle-type	267 (82.41)	43 (82.70)	41 (71.93)	45 (84.91)	51 (92.73)	49 (92.45)	38 (70.37)
	*Ascaris suum*	222 (68.52)	18 (34.62)	49 (85.96)	41 (77.36)	48 (87.27)	43 (81.13)	23 (42.59)
	*Strongyloides* spp.	92 (28.40)	19 (36.54)	10 (17.54)	31 (58.49)	8 (14.55)	9 (16.98)	15 (27.78)
	*Trichuris* spp.	152 (46.91)	15 (28.85)	50 (87.72)	17 (32.08)	47 (85.46)	12 (22.64)	11 (20.37)
Platyhelminthes								
	*Fasciola* spp.	15 (4.63)	5 (9.62)	8 (14.04)	0 (0.0)	0 (0.0)	0 (0.0)	17 (31.48)
Protozoa								
	*Eimeria* spp.	87 (26.85)	12 (23.08)	17 (29.82)	19 (35.85)	5 (9.09)	25 (47.17)	9 (16.67)
	*Isospora* spp.	52 (16.05)	5 (9.62)	11 (19.30)	8 (15.09)	6 (10.91)	7 (13.21)	15 (27.78)
	*Iodamoeba* spp.	70 (21.60)	7 (13.46)	19 (33.33)	10 (18.87)	18 (32.73)	5 (9.43)	11 (20.37)
	*Balantidium* spp.	67 (20.68)	12 (23.08)	15 (26.32)	7 (13.21)	11 (20.00)	14 (26.42)	8 (14.81)

**Table-3:** Prevalence of mixed and co-infection of parasites among pigs in Kalasin province.

Study area	No. of pigs sampled	Coinfection with gastrointestinal parasites (%)			

		Single infection	2	3	³ 4
Somdet	52	14 (26.92)	21 (40.38)	9 (17.31)	3 (5.77)
Huai Phueng	57	16 (28.07)	26 (45.61)	7 (12.28)	4 (7.02)
Na Khu	53	18 (33.96)	23 (43.40)	4 (7.55)	3 (5.66)
Kuchinarai	55	6 (10.91)	24 (43.64)	10 (18.18)	5 (9.09)
Khao Wong	53	23 (43.40)	12 (22.64)	5 (9.43)	2 (3.77)
Na Mon	54	17 (31.48)	10 (18.52)	9 (16.67)	5 (9.26)
Total	324	82 (25.31)	124 (38.27)	48 (14.81)	22 (6.79)

## Discussion

GI parasites pose a significant health challenge that impacts the overall productivity of pig farming on a global scale [17, 19–21]. Parasitic infections in pigs are estimated to be the second most critical concern after African swine fever in tropical and subtropical regions [22]. These parasitic infections significantly impede pig production [14, 17, 23], potentially affecting the performance of swine farms [24]. This issue is particularly prominent in developing countries, such as Thailand, where pig production is plagued by significant mortality rates due to various diseases, including parasitic infection [25–27]. GI parasites are divided into three main groups: Trematodes, cestodes, and nematodes, collectively known as helminths. Helminthiasis in pigs is commonly associated with subclinical infections, which can lead to poor feed conversion rate and delay in market weight [1]. Pigs in developing countries are often affected by various intestinal protozoan parasites, including *Cryptosporidium* spp., *Giardia lamblia*, *B. coli*, *Isospora suis*, and *Eimeria* spp. These parasites are common in pig pens and can cause asymptomatic or subclinical infections. Some of these parasites may adversely affect the health of pigs and food quality [23]. Clinical manifestations such as diarrhea and emaciation may be attributed to *Coccidia*, *Oesophastomum* spp., *Trichuris suis*, and *Strongyloides* spp. Both single and mixed infections are associated with high-level clinical signs [28, 29].

In the present study, we successfully identified eggs of gastrointestinal helminths and protozoan oocysts. A total of 85.19% of the swine population in farms located in Kalasin province were found to be infected with one or more GI parasites. It should be noted that deworming was not performed in some areas of this study, and there are no previous prevalence data for pigs in these regions. Among the identified parasites, Strongyle eggs were the most common nematode in all swine farms and consistently outnumbered other genetically distinct parasites. These findings align with similar research outcomes reported in numerous studies, further confirming that Strongyles are the most frequently detected parasites in pigs [1, 5, 10, 19, 26]. Notably, Sweden has reported a 64% prevalence of Strongyles in their pig population [5]. The high prevalence of Strongyles can be attributed to four super families of this order, namely, *Strongyloides*, *Trichostrongyloidea*, *Ancylostomatoidea*, and *Metastrongyloidea*, together with 29 genera each with multiple species. In addition, several factors have been found to influence the prevalence of GI parasites. These factors include grazing management practices, the use of anthelmintics, economic conditions, and the level of farmer education [18].

*A. suum*, a prevalent helminth in swine, exhibits varying prevalence rates based on farm management practices and geographical regions [20]. This study revealed that *A. suum* infection had a significantly higher prevalence of 222 pigs (68.52%) compared to other regions such as Nepal (19.8%) [4], Sweden (43%) [5], Nigria (12.5%) [15], India (27.5%) [17], Greece (3.3%-4.6%) [20] and Central Africa (3.84%) [23]. *A. suum* is the most common internal parasite in swine, with similar infection rates in both young and adult pigs [30]. This high prevalence could be attributed to the lack of deworming practices in many farms. In pigs, *A. suum* infection can result in pathological lesions, commonly referred to as white spots on the liver. These lesions occur when *A. suum* larvae invade the liver and then migrate to the lungs and eventually mature into adult worms in the small intestine. The presence of these white spots renders the liver unsuitable for human consumption, leading to commercial losses. In addition, *A. suum* can harm the health of pigs by damaging the intestinal membranes and compromising the immune system. This damage can manifest as clinical signs, such as diarrhea and reduced nutrient digestibility, ultimately affecting growth performance [31]. Although *A. suum* is a naturally occurring nematode parasite of pigs, it can also infect humans [32]. The potential of *A. suum* to infect humans is attributed to the shared protein molecules it possesses with *Ascaris lumbricoides*, the definitive natural host that affects approximately 1.2 billion people worldwide [20, 32]. Cooccurrence of *A. lumbricoides* and *A. suum* in the same environment creates two host-associated transmission patterns. This zoonotic relationship implies that pigs act as a reservoir for human infection [10]. A molecular analysis confirmed the presence of shared cytochrome c oxidase subunit 1 (cox1) haplotypes between *Ascaris* from humans and pigs, further supporting the possibility of cross-infection between the two species. Therefore, strict management practices on pig farms are essential not only to protect pig health but also to protect human populations from *A. suum*-induced ascariasis because pigs may serve as potential reservoir hosts for human infection [20].

In the present study, *T. suis* was found to be the third most common infection in 152 pigs (46.91% of cases). This prevalence differed significantly from other research studies where the infection rate was reported to be very low; 1.04% [27], 2.5% [20], 10%–11.5% [5, 26, 29], and 20% [21]. The relatively higher prevalence of *T. suis* in this study could be attributed to the presence of pig pens with soil floors in many farms, which increases the risk of infection. In addition, the lack of deworming in many farms may have contributed to a higher prevalence. Some specific regions, such as Southwest Nigeria (12.2%) [11], also showed notably high prevalence rates of *T. suis*. Infection with *T. suis* can lead to clinical symptoms in pigs, such as diarrhea, anorexia, anemia, poor growth, dehydration, and emaciation. However, the severity of symptoms is often associated with the infective dose or coinfection with bacterial enteritis [33], resulting in significant economic losses in the swine industry [34]. It should be noted that *T. suis* is related to *Trichuris trichiura*, which affects approximately 795 million people. Molecular analysis using the internal transcribed spacer 2 (ITS-2) region of sympatric worms and the ITS region of eggs collected from non-human primates and pigs indicated that *T. suis* infections can be zoonotic, despite the two hosts harboring different species of *Trichuris*. Experiments have confirmed that *Trichuris* spp. can cross-infect both pigs and humans [20]. However, it should be noted that the parasite is non-pathogenic in humans [34]. In this study, *Strongyloides* spp. exhibited a prevalence rate of 28.40%, which was higher than those reported in Indonesia (19%) [21], Central Africa (4.4%) [23], Nan Thailand (2.5%) [26], and Mumbi, India (0.74%) [29]., This parasite is particularly important for suckling piglets because it lives in the small intestine and can lead to clinical symptoms such as diarrhea and dehydration. In cases of severe infection, piglets around 10–14 days old often succumb to the infection, whereas those who survive may experience stunted growth. It should be noted that *Strongyloides ransomi* is not considered zoonotic in pigs [21]. *Iodamoeba butschlii* and *I. suis* are often referred to as *C. suis* (synonymous with *I. suis*) [20]. In the present study, *I. suis* was detected at a prevalence of 16.05%, which was higher than the 6% reported in prior studies in Greece [19], but lower than that reported in Sweden (60%) [5], Central Africa (65.38%) [23] and Nan province, Thailand (25.7%) [26]. *I. suis* is a predominant clinical protozoan parasite in pigs that can lead to transient diarrhea, often caused by secondary pathogens such as bacteria and viruses, resulting in weight loss and increased management costs [3]. *I. suis* infections are a major cause of diarrhea in suckling pigs, typically detected at around 8–10 days of age, but older piglets may also be affected [5]. In addition, *I. suis* may lead to changes in intestinal epithelium and gut microbiota, resulting in reduced absorption of nutrients. Asexual and sexual reproduction of *I. suis* occurs in the epithelium of the small intestine. This protozoan is the primary pathogen that induces diarrhea, with bacteria and viruses causing clinical symptoms [35]. Protozoan infections significantly impact suckling piglets because their primary immune response is insufficient for protection. Infected piglets often show poor growth and need to be removed from the herd and treated with drugs. The timing of drug administration should be consistent with the protozoan life cycle. The 3^rd^ day of piglet life is the optimal time for antiprotozoal medication [3]. Farrowing units are a major source of infection in newborn piglets, which highlights the need for hygienic measures in farrowing pens. *Eimeria* spp. were detected at a prevalence of 26.85% or 87 pigs in this study, which is lower than that in Sweden (64%) [5], Indonesia (78%) [21], and Romania (63.2%) [36] but similar to that in Nan province, Thailand (25.7%) [26]. These protozoa are commonly found in adult pigs but may affect pigs of different ages. Infections are typically subclinical, but heavy infections can lead to diarrhea in piglets, and poor sanitation is associated with heavy infection. In severe cases, pigs may die due to dehydration (water loss ranging from 10% to 59%) [21]. Pigs can be re-infected without clinical symptoms, and this protozoan can spread in the surrounding environment. *Balantidium* spp. was found at a prevalence of 20.86% in this study, which was lower than in Greece (37.8%) [20], Indonesia (79%) [21], Central Africa (76.9%) [23], Romania (70.31%) [36] and Columbia (42%) [37]. This protozoan may cause balantidiosis and is a zoonosis capable of infecting both humans and animals via the fecal-oral route. In swine, it is typically subclinical and resides in the lumen of the cecum and colon. Heavy infections may lead to diarrhea and abdominal discomfort. Factors contributing to infection include climatic conditions, sanitation, and community culture. Urban populations living near pig pens are at risk of transmission of balantidiosis, and veterinarians, animal handlers, and butchers also face a higher risk of infection [21]. In this study, *Iodamoeba buchii* was reported at a prevalence of 21.60%. This zoonotic parasite may be transmitted through contaminated food or water from swine feces. Although *I. buchii* is generally considered nonpathogenic in humans, *I. buchii* is a common intestinal ameba in swine, humans, and apes [37]. In addition, *Fasciola* spp. was detected in this study at a prevalence of 4.63%, similar to that reported in Italy (4.37%) [38], but lower than that reported in Nepal (9%) [39], Nigeria (9.3%) [40], and China (1.3%) [41]. This trematode parasite has rarely been reported and may infect pigs if pig pens are located close to cattle areas, allowing cross-species transmission. Adult pigs are usually resistant to this parasite due to the fibrous nature of the liver parenchyma and immune responses that act as mechanical barriers. However, suckling piglets are more susceptible than adults to *Fasciola* infection [39].

Pig farmers generally use either ivermectin or fenbendazole to combat GI parasites. However, in spite of these efforts, parasites continue to pose significant challenges on pig farms, suggesting that anthelmintic resistance may emerge [42]. The routine use of anthelmintic drugs contributes to the risk factors associated with severe anthelmintic resistance in nematode parasites, particularly *Oesophagostomum* spp. of the Strongyle order. In addition, it has been reported that toltrazuril is less effective against *I. suis*. Effective control of parasites in pig farms can be achieved through a combination of anthelmintic drugs and strategic hygiene and biosecurity measures [5]. The transmission of parasites in pigs is either direct or can contaminate food by ingestion. In addition, environmental conditions significantly impact the level of infection in animals. The high prevalence of GI parasites is often associated with poor hygiene practices, specific climatic conditions, and the transmission of parasites. In small farms, pig pens may not be regularly cleaned, deworming may not take place often or infrequently, and pigs may be undernourished or receive inadequate nutrition. Conditions conducive to the proliferation of parasite infections include high rainfall, high temperatures, and high humidity [23]. In this study, most small farms in Kalasin shared similar conditions, including temperature and humidity levels in the region, as well as parasite control practices. The high prevalence observed in Kalasin province suggests a lack of hygienic and sanitary conditions in these pig farms, which may contribute to the propagation and transmission of parasites among animals and humans.

## Conclusion

In this study, high prevalence of gastrointestinal parasites was observed in pigs and it might affect economic losses in pig production. The presence of *Fasciola* spp. poses a significant public health risk. Strongyle-type parasites dominate among GI parasites in this region. This study highlights the significant prevalence of GI parasites in pigs within Kalasin province, suggesting that this area could serve as a potential reservoir for parasites, posing a risk of future outbreaks. To reduce parasite infections, it is recommended to use deworming every 6 months for pigs, to ensure proper management of the pen and to maintain dry conditions in the pig-rearing area.

## Authors’ Contribution

ST: Planned and designed the experiment and drafted and revised the manuscript. SP and CA: Data analysis and drafted the manuscript. KT: Data analysis and the fieldwork. All authors have read, reviewed, and approved the final manuscript.

## References

[1] Atawalna J, Attoh-Kotoku V, Folitse1 R.D, Amenakpor C (2016). Prevalence of gastrointestinal parasites among pigs in the Ejisu Municipality of Ghana. Sch. J. Agric. Vet. Sci.

[2] Olaniyi A.J (2014). Public health implication of gastrointestinal parasites of pigs in Kwara State, Nigeria. J. Environ. Res. Manage.

[3] Pradella B, Molosse K.F, Menin M, Matzembacker B, Biondo N, Vanazzi D.L, Baldasso N, Bennemann P.E, Prestes A, Camillo G (2020). Occurrence of gastrointestinal parasitic diseases of swine in different production phases in commercial pig farms from the State of Santa Catarina, southern Brazil. Arq. Bras. Med. Vet. Zootec.

[4] Sah R.P (2018). Prevalence of common gastrointestinal nematode parasites in pigs based on different altitudes and seasons in Dhankuta and Sunsari districts of Nepal. Nepal. J. Agric. Sci.

[5] Pettersson E, Sjolund M, Doreac F.C, Lind E.O, Grandi G, Jacobson M, Höglund J, Wallgren P (2021). Gastrointestinal parasites in Swedish pigs:Prevalence and associated risk factors for infection in herds where animal welfare standards are improved. Vet. Parasitol.

[6] Chaisiri K, Aueawiboonsri S, Kusolsuk T, Dekumyoy P, Sanguankiat S, Homsuwan N, Peunpipoom G, Okamoto M, Yanagida T, Sako Y, Ito A (2017). Gastrointestinal helminths and *Taenia* spp. in parenteral tissues of free-roaming pigs (*Sus scrofa indicus*) from hilltribe village at the western border of Thailand. Trop. Biomed.

[7] Visetnoi S, Nelles W (2023). Can organic pork help achieve sustainable development Goals in Thailand?. Agriculture.

[8] Krishna Murthy C.M, Ananda K.J, Adeppa J, Satheesha M.G (2016). Studies on gastrointestinal parasites of pigs in Shimoga region of Karnataka. J. Parasit. Dis.

[9] Tumusiime M, Ntampaka P, Niragire F, Sindikubwabo T, Habineza F (2020). Prevalence of swine gastrointestinal parasites in Nyagatare District, Rwanda. J. Parasitol. Res.

[10] Agustina K.K, Swacita I.B.N, Oka I.B.M, Dwinata I.M, Traub R.J, Cargill C, Damriyasa I.M (2017). Reducing zoonotic and internal parasite burdens in pigs using a pig confinement system. Vet. World.

[11] Sowemimo O.A, Asaolu S.O, Adegoke F.O, Ayanniyi O.O (2012). Epidemiological survey of gastrointestinal parasites of pigs in Ibadan, Southwest Nigeria. J. Public Health Epidemiol.

[12] Vlaminck J, Levecke B, Vercruysse J, Geldhof P (2014). Advances in the diagnosis of *Ascaris suum* infections in pigs and their possible applications in humans. Parasitology.

[13] Idika I.K, Njoga U.J, Ezeh I.O, Iheagwam C.N, Ezenduka E.V, Njoga E, Onah D.N (2017). Re-evaluation of porcine cysticercosis in Nsukka area of Enugu State, Nigeria. Asian Pac. J. Trop. Dis.

[14] Abonyi F.O, Njoga E.O (2020). Prevalence and determinants of gastrointestinal parasite infection in intensively managed pigs in Nsukka agricultural zone, Southeast, Nigeria. J. Parasit. Dis.

[15] Nathaniel A.O, Anyika K.C, Frank M.C, Jatau J.D (2017). Prevalence of Gastro-intestinal parasites in pigs in Jos South local government area of plateau state, Nigeria. Haya Saudi J. Life Sci.

[16] Mpofu T.J, Nephawe K.A, Mtile B (2020). Prevalence of gastrointestinal parasites in communal goats from different agro-ecological zones of South Africa. Vet. World.

[17] Kaur M, Singh B.B, Sharma R, Gill J.P.S (2017). Prevalence of gastrointestinal parasites in pigs in Punjab, India. J. Parasit Dis.

[18] Thanasuwan S, Piratae S, Tankrathok A (2021). Prevalence of gastrointestinal parasites in cattle in Kalasin Province, Thailand. Vet. World.

[19] Roesel K, Dohoo I, Baumann M, Dione M, Grace D, Clausen P.H (2017). Prevalence and risk factors for gastrointestinal parasites in small-scale pig enterprises in Central and Eastern Uganda. Parasitol. Res.

[20] Symeonidou I, Tassis P, Gelasakis A.I, Tzika E.D, Papadopoulos E (2020). Prevalence and risk factors of intestinal parasite infections in Greek swine farrow-to-finish farms. Pathogens.

[21] Widisuputri N.K.A, Suwanti L.T, Plumeriastuti H (2020). A survey for zoonotic and other gastrointestinal parasites in pig in Bali Province, Indonesia. Indonesia. J. Trop. Infect. Dis.

[22] Permin A, Yelifari L, Bloch P, Stenhard N, Hansen N.P, Nansen P (1999). Parasites in cross-bred pigs in the Upper East region of Ghana. Vet. Parasitol.

[23] Maganga G.D, Kombila L.B, Boundenga L, Kinga I.C.M, Obame-Nkoghe J, Tchoffo H, Gbati O.B, Awah-Ndukum J (2019). Diversity and prevalence of gastrointestinal parasites in farmed pigs in Southeast Gabon, Central Africa. Vet. World.

[24] Tachawarung W, Boonchuen S, Boonshuya C, Saiwichai T (2015). Prevalence and Associated Factors of Swine Gastrointestinal Parasite Infections in Photharam District, Ratchaburi Province. Kasetsart University Kamphaeng Saen Campus Held National Academic Conferences 12^th^.

[25] Soderberg R, Lindahl J.F, Henriksson E, Kroesna K, Ly S, Sear B, Unger F, Tum S, Nguyen-Viet H, Ström Hallenberg G (2021). Low prevalence of cysticercosis and *Trichinella* infection in pigs in rural Cambodia. Trop. Med. Infect. Dis.

[26] Unjit K, Wattanamethanont J, Mohkaew K, Ngamjiteua S, Tablerk P, Hinjoy S (2012). Prevalence and risk factors of internal parasite among pigs in Nan province during January-April 2011. Thai NIAH eJournal.

[27] Moonsan P, Moonsan Y, Saokhieo J, Juntra P, Sangpan T (2018). Prevalence and quantity of gastrointestinal parasites in sows of Ban Na Chak Wai farmers in Phitsanulok Province *Mahanakorn Vet*. Med.

[28] Bauri K, Ranjan R, Deb A.R, Ranjan R (2012). Prevalence and sustainable control of *Balantidium coli* infection in pigs of Ranchi, Jharkhand, India. Vet. World.

[29] Dadas S, Mishra S, Jawalagatti V, Gupta S, Vinay T.S, Gudewar J (2016). Prevalence of gastro-intestinal parasites in pigs (*Sus scrofa*) of Mumbai region. Int. J. Sci. Environ. Technol.

[30] Nsoso S.J, Mosala K.P, Ndebele R.T, Ramabu S.S (2000). The prevalence of internal and external parasites in pigs of different ages and sexes in Southeast District, Botswana. Onderstepoort J. Vet. Res.

[31] Lee S, Alkathiri B, Kwak D, Lee S.M, Lee W.K, Byun J.W, Lee S.H (2022). Distribution of gastrointestinal parasitic infection in domestic pigs in the Republic of Korea:Nationwide survey from 2020–2021. Korean J. Parasitol.

[32] Tomass Z, Imam E, Kifleyohannes T, Tekle Y, Weldu K (2013). Prevalence of gastrointestinal parasites and *Cryptosporidium* spp. in extensively managed pigs in Mekelle and urban areas of Southern zone of Tigray region, Northern Ethiopia. Vet. World.

[33] Pittman J.S, Shepherd G, Thacker B.J, Myers G.H (2010). *Trichuris suis* in finishing pigs:Case report and review. J. Swine Health Prod.

[34] Muramatsu R, Sato R, Onuma N, Sasai K, Shibahara T, Matsubayashi M (2020). Molecular Identification of *Trichuris suis* worms and eggs in pig feces, infected intestines, and farm environments in Japan. J. Agric. Res. Q.

[35] Niestrath M, Takla M, Joachim A, Daugschiesa A (2002). The role of *Isospora suis* as a pathogen in conventional piglet production in Germany. J. Vet. Med. B Infect. Dis. Vet. Public Health.

[36] Băie M.H, Boros Z, Gherman C.M, Spînu M, Mathe A, Pataky S, Lefkaditis M, Cozma V (2022). Prevalence of swine gastrointestinal parasites in two free-range farms from Nord-West Region of Romania. Pathogens.

[37] Mendoza-Gómez M.F, Pulido-Villamarín B.A, Barbosa-Buitrago A, Aranda-Silva M (2015). Presence of gastrointestinal parasites in swine and human of four swine production farms in Cundinamarca-Colombia. Rev. MVZ Córdoba.

[38] Capucchio M.T, Deborah C, Miriam R, Vincenzo A, Amedeo T, Alessandro L, Stefano A, Eleonora S.F, Bruno D, Franco G (2009). Natural trematode infestation in feral Nebrodi Black pigs:Pathological investigations. Vet. Parasitol.

[39] Adhikari R.B, Adhikari Dhakal M, Thapa S, Ghimire T.R (2021). Gastrointestinal parasites of indigenous pigs (*Sus domesticus*) in South-Central Nepal. Vet. Med. Sci.

[40] Bernard A.N, Daminabo V, Ekam E, Okonkwo E, Nwuzo A, Afiukwa F, Agah M (2015). Prevalence of intestinal parasites in faecal droppings of swine in Pankshin Urban, Pankshin local government area, Plateau state, Nigeria. Am. J. Life Sci.

[41] Boes J, Willingham A, Fuhui S, Xuguang H, Eriksen L, Nansen P, Stewart T (2000). Prevalence and distribution of pig helminths in the Dongting Lake region (Hunan province) of the People's republic of China. J. Helminthol.

[42] Tiwari K.P, Chikweto A, Belot G, Vanpee G, Deallie C, Stratton G, Sharma R.N (2009). Prevalence of intestinal parasites in pigs in Grenada. WIVJ.

